# Construct and concurrent validity of test of infants’ daily living activities

**DOI:** 10.3389/fneur.2026.1662168

**Published:** 2026-03-17

**Authors:** Martyna Franecka, Małgorzata Domagalska-Szopa, Andrzej Szopa

**Affiliations:** 1 Institute of Health Sciences, University of Opole, Opole, Poland; 2 Departament of Developmental Age, Medical University of Silesia, Katowice, Poland; 3 Departament of Physiotherapy, Medical University of Silesia, Katowice, Poland

**Keywords:** diagnostics, disability and health, infancy, motor development, scale

## Abstract

**Background:**

Delays in psychomotor development in children could be an early indicator of elevated risk for developmental disorders. Early functional diagnostics enables the identification of abnormalities in various developmental domains and contributes to the initiation of specialized neurodevelopmental diagnostics. The objective of this study was to assess the construct and concurrent validity of the Test of Infants’ Daily Living Activities. The focus was on identifying the construct validity of the Test of IDLA, demonstrating the concurrent validity of the Test of IDLA in relation to the Alberta Infant Motor Scale, as well as determining the predictive value of the Test of IDLA in assessing the development of postural and motor control in infants.

**Methods:**

The study included 357 children aged 1–18 months. Each child was thoroughly evaluated using the AIMS scale, in accordance with the methodology of the diagnostic tool’s usage, and the Test of IDLA, which involved a postural control assessment sheet and a motor control evaluation sheet.

**Results:**

Comparison of Test of IDLA results with the reference AIMS scale demonstrated a statistically significant, positive correlation, with Spearman’s rank correlation coefficient rho = 0.72, *p* < 0.0001. A very strong relationship between both scales was also indicated by the Cramer’s V result = 0.78 and χ^2^ (1, *n* = 357) = 217.518. The area under the ROC curve value (AUC = 0.928) indicated very good diagnostic capability of the Test of IDLA. The narrow confidence interval (0.897–0.953) and low standard error (0.0147) emphasized the stability and repeatability of Test of IDLA results under clinical conditions.

**Conclusion:**

The Test of IDLA demonstrates high concordance with the reference AIMS scale in identifying motor developmental delays in children, in the domains of postural control and motor control. These findings indicate the utility of the Test of IDLA in the screening identification of neurodevelopmental disorders.

## 1 Introduction

Psychomotor developmental delay in the infant period could indicate a potential risk of the occurrence of developmental disorders in children (1). Early assessment of psychomotor development in infants allows for the recognition of delays, deficits, or disorders in its various areas, which enables the identification of infants in whom specialized neurodevelopmental diagnostics should be undertaken (2). The latest global recommendations concerning the detection of risk for neurodevelopmental disorders in young children (primarily cerebral palsy; CP) recommend supplementing basic diagnostics based on neuroimaging studies (primarily magnetic resonance imaging; MRI) with an assessment of infant motor development using validated and standardized developmental scales (3).

Currently, in clinical practice, several well-documented developmental classifications are used to detect psychomotor developmental disorders, such as: the Standardized Motor Assessment (MAI) and Alberta Infant Motor Scale (AIMS), Neuro Sensory Motor Development Assessment (NSMDA), Test of Infant Motor Performance (TIMP), Peabody Developmental Motor Scales (PDMS-GM), Hammersmith Infant Neurological Examination (HINE), Developmental Assessment of Young Children (DAYC), and Prechtl’s General Movements Assessment (GMA) (4).

A review of evidence-based research results regarding the prognostic value of diagnostic tools used for recognizing and predicting CP in newborns and infants demonstrated that for younger infants (i.e., those who have not yet reached 5 months of corrected age), the tools with the greatest capacity for detecting developmental risk are magnetic resonance imaging (MRI; sensitivity 86–89%), Prechtl Qualitative Assessment of General Movements (GMA; sensitivity 98%), and Hammersmith Infant Neurological Examination (HINE; sensitivity 90%). For older infants (i.e., those who have reached 5 months of corrected age), the most predictive tools for detecting developmental risk are magnetic resonance imaging combined with assessment using the Hammersmith Infant Neurological Examination (HINE) and Developmental Assessment of Young Children (DAYC). However, if performing an MRI on an infant is not safe or not feasible, diagnostics should be expanded with additional clinical diagnostics. For younger infants (i.e., up to 5 months of corrected age), conducting the Test of Infant Motor Performance (TIMP) is recommended, while for older infants (i.e., after 5 months of corrected age), conducting the Developmental Assessment of Young Children (DAYC) is advised. Additionally, in both cases it is recommended to conduct motor development assessment using the Alberta Infant Motor Scale (AIMS) or the Neuro Sensory Motor Development Assessment (NSMDA) (4).

Challenges in early recognition of motor developmental disorders in infants in our country (Poland) include both the small percentage of MRI examinations performed on children under two years of age, as well as the lack of tools for assessing motor development in young children that have been translated into Polish and culturally validated.

To address the problem of the lack of a validated tool for assessing child motor development, the *TEST OF INFANTS’ DAILY LIVING ACTIVITIES (Test of IDLA)* was developed on the initiative of the Polish professional self-government of physiotherapists (National Council of Physiotherapists, 1st term; KRF) by the Thematic Team for Quality and Monitoring of the Physiotherapy Process. The first version of the Test of IDLA was developed under the title “Guidelines of the National Council of Physiotherapists for functional assessment and medical documentation management for developmental age patients (0-7)” and was published in 2020 (5), while an update which consisted of minor specifications was introduced in 2023, and this development was included in a broader publication encompassing “Guidelines of the National Council of Physiotherapists for providing healthcare services in physiotherapy and their description in medical documentation” (6).

The *TEST OF INFANTS’ DAILY LIVING ACTIVITIES (Test of IDLA)* involves the observation and point assessment of spontaneous child activity in the following domains: (1) postural control (ability to assume and maintain specific positions); (2) motor control (gross motor skills); and observation combined with obtaining responses to questions posed to parents/caregivers of the child regarding: (3) dexterity (fine motor skills); and (4) self-care (social and emotional development), specifically defined and illustrated in appendices created expressly for this purpose. The assessment of spontaneous activity is conducted separately for children aged 0–24 months (part 1) and 2–7 years (part 2). Points are awarded according to the following criteria (5):

1. In the domain of postural control: 2 points - when the child independently assumes and maintains position; 1 point - when the child assumes position with physical assistance from the examiner and independently maintains it; 0 points - when the child cannot assume position (even with assistance) or maintain it.
2. In the domains of motor control, dexterity, and self-care: 2 points - when the child independently performs a motor activity; 1 point - when the child performs the motor activity with assistance from the examiner; 0 points - when the child does not perform the motor activity (even with assistance).

The measure of functional assessment here is the value of individual activity indicators (AI) determined in each domain (5, 6). The activity indicators are calculated based on the sum of points obtained in the examination and the child’s age, separately for children aged 0–24 months and 2–7 years, and separately for each domain. Areas of child development can therefore be assessed independently, so that examiners can evaluate only the domains that are of interest to them or test all four areas when measurement of general developmental level is desired, calculating the mean result from all domains.

The activity indicators for children aged 0–24 months - according to the following formula:


$$\mathrm{AI}=\frac{\mathrm{sum}\;\text{of}\;\mathrm{all}\;\text{scores obtained}}{{\text{child}}^{’}s\;\mathrm{ag}e_{\left(\text{completed months};\text{corrected}\;\mathrm{age}-\text{adjusted for prematurity}\right)}}$$

Each activity indicator reflects the current level of a child’s activity in a given area (activity indicator in a specific domain) or in terms of general developmental level (global activity indicator; mean value from 4 domains). This scale is constructed so that activity indicator values oscillate within the range 0–4. The higher the indicator value, the higher the child’s activity level. Interpretation of activity indicator AI: 0 ≤ AI<1 Very low; 1 ≤ AI<2 Low; 2 ≤ AI<3 Average; 3 ≤ AI≤4 High. The Test of IDLA was developed as a tool for observational assessment of spontaneous activity. The test begins with an observation and scoring of skills assigned to the child’s (appropriate) age range. Initially, skills from the completed month of life are assessed (in the case of children born before 37 weeks’ gestation, it is corrected age). For example, for a child who has turned 3 months but has not yet begun the 4th month, the appropriate skill range is “3 months of life” and their age in months is 3. The requirement for classifying the skills as adequate for the child’s age in each assessed area (domain) is obtaining the maximum number of points for each of the 2 specific skills, i.e., 4 points. Then each less mature skill from lower age ranges is assigned 2 points, i.e., a child aged 3 months who obtained 2 × 2 points for skills from the 3-month range is assigned 2 × 2 points for skills from the 2-month range and 2 × 2 from the 1-month range. Such a child obtains a total of 12 points in a given area, and their activity indicator equals 12 points ÷ 3 months = 4. In situations when a child, instead of obtaining the maximum number of points for age-adequate skills (4 points), obtains 3, 2, 1, or 0 points, we assess their skills in accordance with the requirements for lower maturity levels until obtaining the threshold condition, i.e., 2 × 2 points. Then we assign 2 × 2 points to each level below age and add to the point sum those obtained for skills from higher levels. For example, when a child aged 3 months obtains 1 point for each skill from the 3-month range (i.e., 2 points), and for skills from the 2-month level meets threshold conditions, i.e., 2 × 2, then their point sum equals: 4 points (for 2 months) + 4 points (2 × 2) assigned for 1 month + 2 points (2 × 1 for 3 months) = 10 points, their activity indicator is 10 points ÷ 3 months = 3.3. By design, the Test of IDLA does not assess skills from ranges above the child’s age.

**Figure 1:**
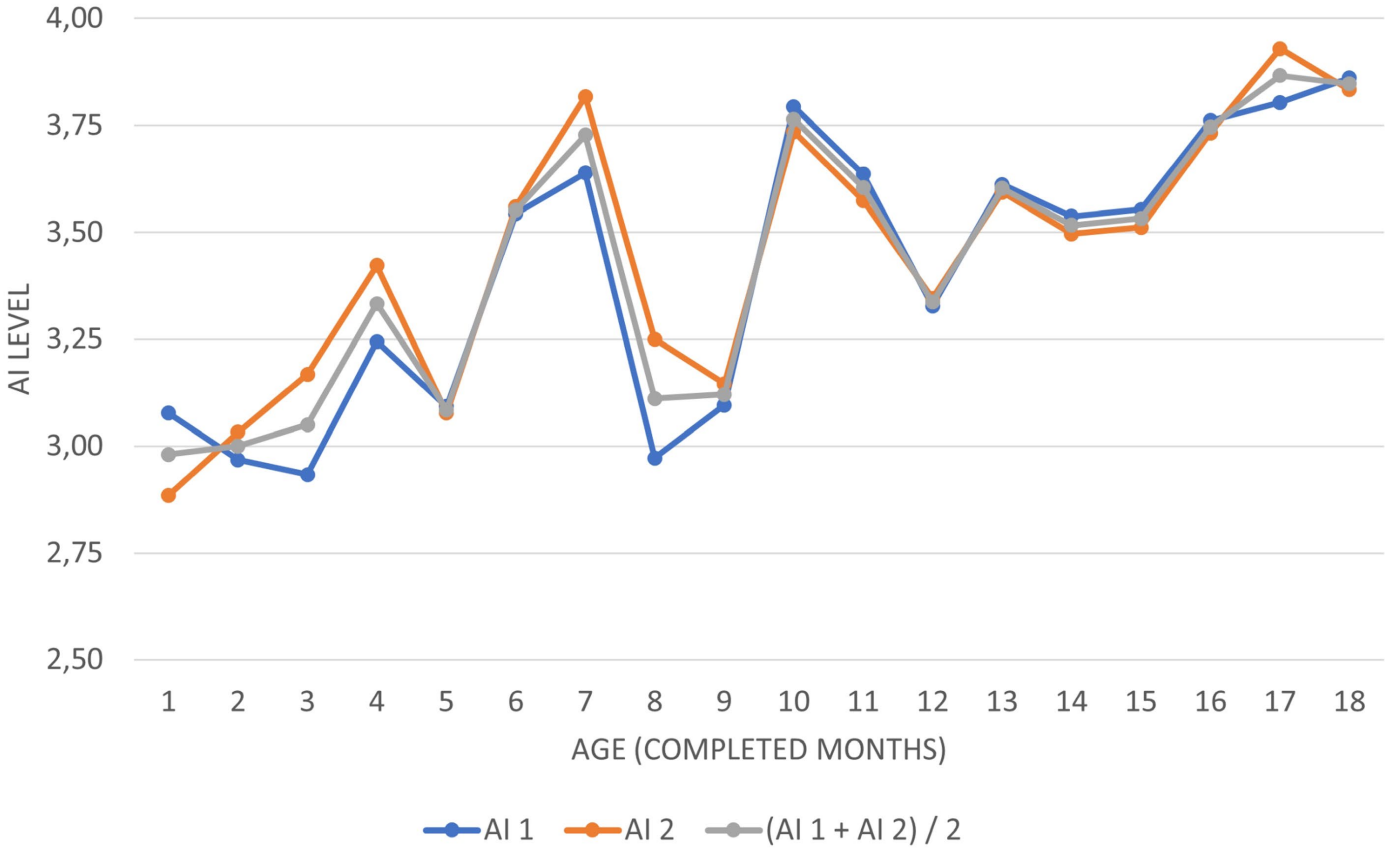
The course of changes in activity indicators of the examined group of infants in the domains of AI 1, AI 2, and AI 1 & AI 2.

Including the child’s current age in the activity indicator calculations allows for not only a quantifiable evaluation of the child’s activity level and a comparison of activity levels among children of different ages, but also precise tracking of changes in the child’s activity level over time, i.e., affected by physical and psychomotor development, disturbed CNS maturation, or applied neurodevelopmental therapy (Figure 1).

The Test of IDLA is a simple method for assessing child development, and conducting the assessment/evaluation in one area takes only several minutes. Its application in basic physiotherapy practice, both for functional assessment needs and physiotherapy documentation management, does not require any specialized training in this area. Individual skills have been listed on 4 purposely developed assessment cards and additionally explained in both publications listed above, in the form of photographic documentation. The IDLA scale is also available free of charge for every physiotherapist with the right to practice the profession, in the electronic FINEZJO application (https://app.finezjo.pl/logowanie), which significantly shortens assessment documentation time and enables obtaining various forms of graphic documentation, making it extremely appealing.

The research project, which aimed at (1) demonstrating construct validity of the IDLA scale; (2) demonstrating concurrent validity of the IDLA scale in relation to the Alberta Infant Motor Scale (AIMS); and (3) demonstrating predictive value of the Test of IDLA in psychomotor development assessment; as well as (4) repeatability and (5) reliability of IDLA assessment, was launched in 2023. The studies conducted within this work are part of the aforementioned project and concern construct validation of the Test of IDLA, i.e., assessment of psychomotor development in infants in the area of postural abilities (postural control domain) and motor skills (motor control domain) and are part of a broader research project.

The objective of the conducted studies was:

(1) to recognize the construct validity of the Test of IDLA; (2) to demonstrate the concurrent validity of the Test of IDLA in relation to the Alberta Infant Motor Scale (AIMS). The decision was made to conduct comparative studies relative to the AIMS scale as it is the most frequently used, normalized, translated into Polish, and culturally validated tool utilized for motor assessment of infants (7). Despite the very simple construction of the IDLA scale and the short time required to conduct the Test of IDLA compared to the very detailed and lengthy AIMS assessment, a research hypothesis was posed regarding a high correlation of Test of IDLA results in assessing the development of postural and motor control in infants with the results of motor development assessment in infants aged 0–18 months obtained using the AIMS scale.

## 2 Materials and methods

The study obtained approval from the Thematic Team for Ethics in Scientific Research of Physiotherapists at the Polish Chamber of Physiotherapists by resolution dated June 29, 2022 (approval no. 15/52) and is consistent with the Declaration of Helsinki. The study was self-funded. Each parent was thoroughly informed about the study objectives, the nature of collected data, the inclusion and exclusion criteria, and received information about the possibility of withdrawing from the project, after which they expressed informed, written consent for their children’s participation in the research.

### 2.1 Materials

The study was carried out on 357 children aged 1–18 months, including 175 girls and 182 boys, who were referred for early physiotherapeutic intervention by a pediatrician. Inclusion criteria included: (1) age below 18 months and (2) informed consent of parents/legal guardians of the child for participation in the study. Participation was excluded if: (1) the infants had major congenital anomalies and genetic syndromes; (2) they were clinically unstable; (3) they recently suffered injuries or undergone surgical procedures; (3) they had an infection or inflammation during examination; and (4) they were newborns whose parents/legal guardians had not approved the examination. The characteristics of the examined subjects are presented in the Table 1. To ensure standardized assessment conditions, all evaluations were conducted at a single, dedicated research facility in Opole, Poland. However, to mitigate selection bias and enhance the representativeness of the sample for the region, recruitment was conducted at a population-based level across the entire Opole Voivodeship. The recruitment strategy was multifaceted. We collaborated with a network of paediatricians working in both public and private healthcare institutions across the voivodeship, who referred potential participants. Informational leaflets and posters were distributed in numerous primary care paediatric clinics and children’s centres throughout the region. Targeted social media advertisements were also used to reach parents of infants within the Opole Voivodeship.

**Table 1:** Characteristics of examined children (*n* = 357).

Parameter	M ± SD	Min-Max	Me	Q1	Q3	Q1-Q3	IQR
Apgar	9.69 ± 0.86	3–10	10	10	10	10–10	0
Birth weight (g)	3385.14 ± 527.14	1780–4780	3410	3100	3720	3100–3720	620
Child’s Age (months)	59 ± 5.22	1–18	8	4	13	4–13	9
Gestational Age (hbd)	38.90 ± 1.40	34–41	39	38	40	38–40	2
Sex: *n* (%); SE%							
Male	182 (51); 4						
Female	175 (49); 4						
Mode of delivery: *n* (%); SE%							
vaginal delivery	166 (46); 4						
cesarean section	191 (54); 4						
Preterm: *n* (%); SE%	26 (7); 5						
In term: *n* (%); SE%	331 (93); 1						

### 2.2 Methods

The study protocol consisted of 3 parts conducted in the following order: (1) an interview with the parents/legal guardians of the child, (2) an assessment of the child using the AIMS scale, and (3) an assessment of the child using the Test of IDLA.

The interview conducted with the parents/legal guardians of the child aimed to collect detailed information regarding the child’s health status in the prenatal, perinatal, and postnatal periods. It included: demographic data including sex and age of the child given in months, as well as perinatal data: Apgar score, birth weight given in grams, gestational week at which delivery occurred, type of delivery (SVD – spontaneous vaginal delivery, CS – cesarean section).

The study utilized the Polish version of the Alberta Infant Motor Scale (AIMS). The examination methodology was consistent with the authors’ recommendations and included observation of the child’s spontaneous motor activities with minimal interference from the examiner, in each of the four subscales: prone position, supine position, sitting position, and standing position. Three components were assessed: weight bearing, posture and anti-gravity movements. For each participant, their least and most mature motor skills were identified and assessed, in relation to the so-called “motor window” of the child’s current motor capabilities. A skill observed and scored as 1 point was recognized as one that appeared during examination according to the linear scheme presented in the graphic material and according to the description contained in the AIMS sheet. For each position below the least mature position observed, in each of the subscales, 1 point was awarded. The final score was calculated by summing up all awarded points, which constituted the total AIMS score (max. 58 pts).

Subsequently, each child was assessed using the Test of IDLA, utilizing the postural control assessment sheet and motor control assessment sheet. The ability to assume and maintain specific positions in the area of postural control, as well as gross motor development in the area of motor control were assessed. Points were awarded as follows: 0 pts - if the child could not assume and maintain position or did not perform the motor activity, 1 pt - if they assumed position with assistance and independently maintained it or when they performed motor activity with assistance, 2 pts - if they independently assumed and maintained position or performed the motor activity without the examiner’s assistance. For all examined subjects, activity indicators AI 1, AI 2, and AI_(1 & 2)_ (domains 1 & 2) were calculated according to the formulas:


$$\mathrm{AI}\;1=\frac{\text{motor control score}}{{\text{child}}^{’}s\;\mathrm{ag}e_{\left(\text{completed months};\text{corrected}\;\mathrm{age}-\text{adjusted for prematurity}\right)}}$$

$$\mathrm{AI}\;2=\frac{\text{postural control score}}{{\text{child}}^{’}s\;\mathrm{ag}e_{\left(\text{completed months};\text{corrected}\;\mathrm{age}-\text{adjusted for prematurity}\right)}}$$

$$AI_{\left(1\&2\right)}=\frac{\mathrm{AI}\;1+\mathrm{AI}\;2}{2}$$

### 2.3 Statistical analysis

Statistical analysis was conducted using MedCalc® Statistical Software version 23.0.6, GPower® version 3.1.9.7, and Microsoft Excel (version 2,412.)

Categorical variables were described as proportions and standard error of proportions. For continuous data, normality of distribution was assessed using the Shapiro–Wilk test. Continuous data with non-normal distribution were presented as median, range from 1st to 3rd quartile, and interquartile range. Face validity and content validity were determined using the ratio of the number of positive opinions to the number of negative opinions. Construct validity was assessed in multiple stages. First, Spearman’s rank correlation was applied to examine the overall correlation of results between the Test of IDLA and AIMS. Subsequently, through the ROC curve analysis, the cutoff point for the Test of IDLA was determined. For Spearman’s rank correlation coefficient, the following interpretation of results was adopted (8): 0.9–1.0: Very strong correlation; 0.7–0.9: Strong correlation; 0.5–0.7: Moderate correlation; 0.3–0.5: Weak correlation; 0.0–0.3: Very weak or no correlation. Subsequently, logistic regression analysis was conducted and the concordance of low/normal results for both tests was examined using the χ^2^ test.

## 3 Results

### 3.1 Descriptive statistics

**Figure 2:**
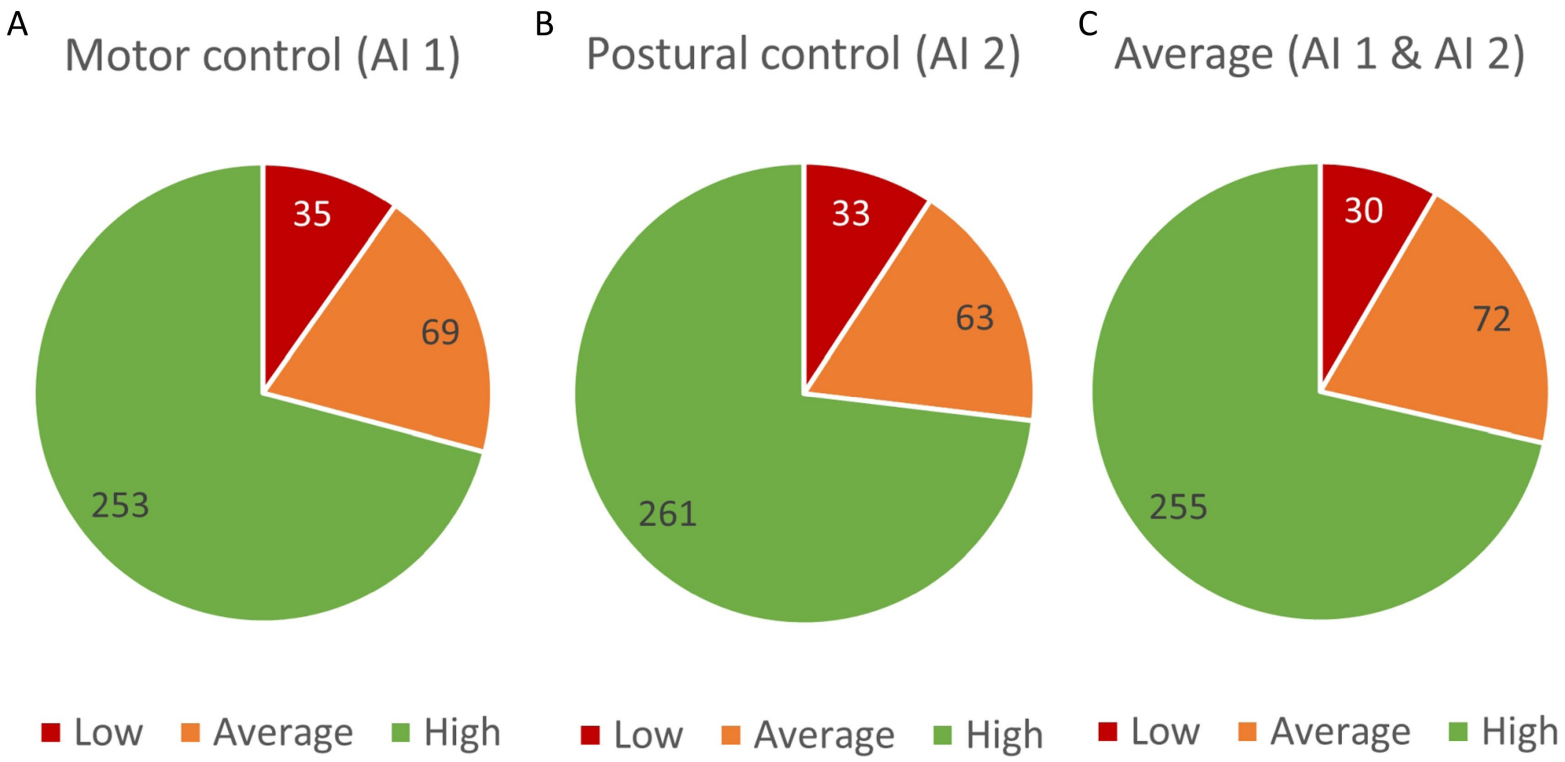
Distribution of examined children according to activity level in the Test of IDLA. **(A)** Motor control domain (AI 1), **(B)** Postural control domain (AI 2), **(C)** Average score across both domains (AI 1 & AI 2).

**Figure 3:**
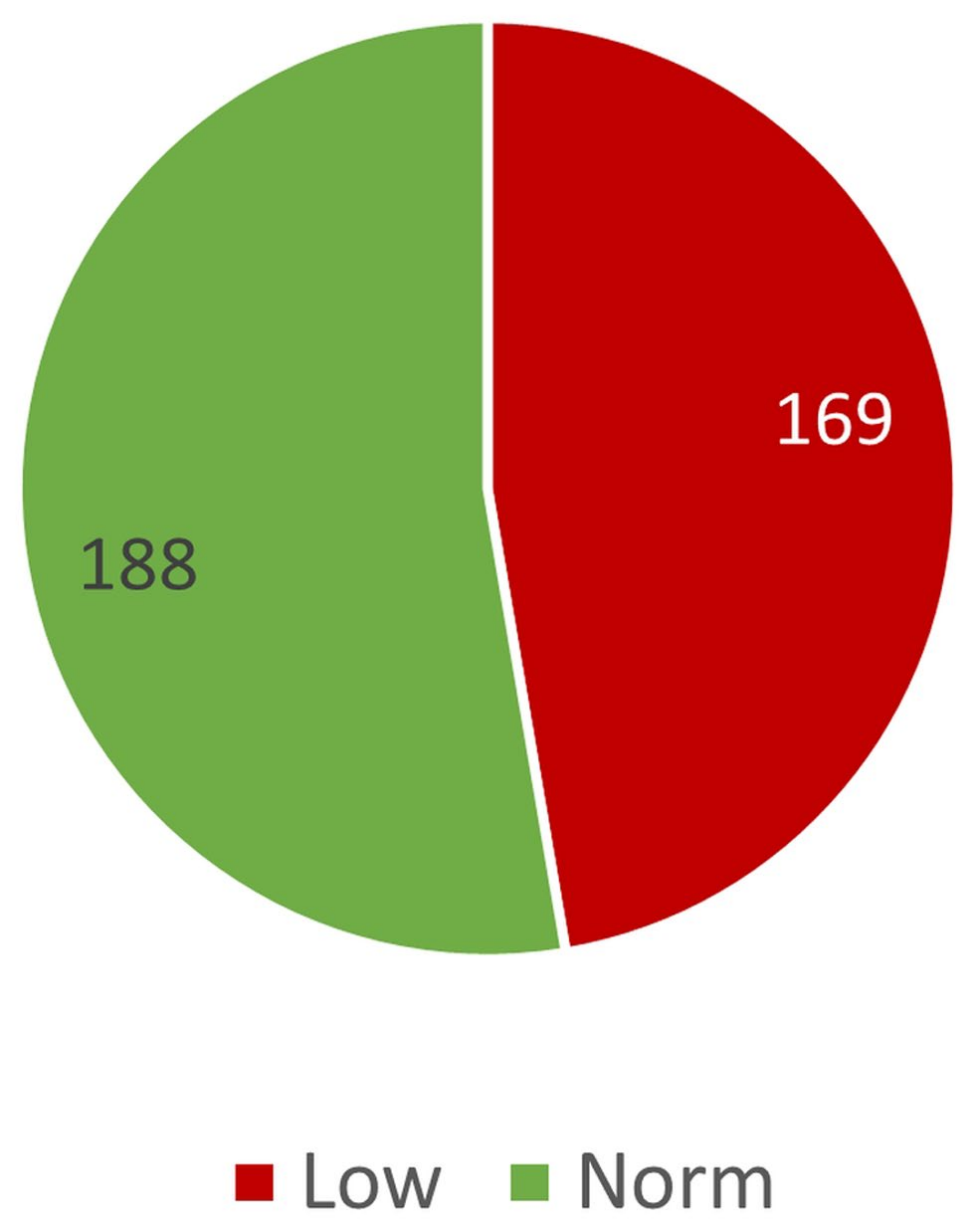
Distribution of examined children according to AIMS test results.

The tables present a compilation of results obtained by study participants in the Test of IDLA (Table 2; Figures 2A–C) and AIMS (Table 3; Figure 3).

**Table 2:** Distribution of examined children according to activity level in the test of IDLA.

Activity index	Activity level	*n*	M ± SD	Min-Max	Me	Q1	Q3	Q1–Q3	IQR
AI 1	Low	35	1.89 ± 0.23	1.20–2.00	1.89	2.00	2.00	2.00–2.00	0.00
	Average	69	2.75 ± 0.22	2.13–3.00	2.75	2.67	2.93	2–67-2.93	0.26
	High	253	3.77 ± 0.31	3.08–4.00	3.77	3.50	4.00	3.50–4.00	0.50
	Total	357	3.37 ± 0.73	0.67–4.00	3.58	3.00	4.00	3.00–4.00	1.00
AI 2	Low	33	1.89 ± 0.26	1.22–2.00	1.89	2.00	2.00	2.00–2.00	0.00
	Average	63	2.73 ± 0.25	2.27–3.00	2.73	2.50	3.00	2.50–3.00	0.50
	High	261	3.79 ± 0.30	3.07–4.00	3.79	3.63	4.00	3.63–4.00	0.37
	Total	357	3.41 ± 0.75	0.67–4.00	3.71	3.00	4.00	3.00–4.00	1.00
AI (1 & 2)	Low	30	1.79 ± 0.32	1.11–2.00	1.79	1.50	2.00	1.50–2.00	0.50
	Average	72	2.77 ± 0.21	2.25–3.00	2.77	2.60	3.00	2.60–3.00	0.40
	High	255	3.76 ± 0.30	3.04–4.00	3.76	3.57	4.00	3.57–4.00	0.43
	Total	357	3.39 ± 0.71	0.67–4.00	3.67	3.00	4.00	3.00–4.00	1.00

**Table 3:** Distribution of examined children according to AIMS test results.

Month	Age	Low	Norm	M ± SD	Min-Max	Me
	*n* (%)					
1	26 (7)	19 (5.3)	7 (2.0)	3.27 ± 1.19	2.00–5.00	3.00
2	31 (9)	21 (5.9)	10 (2.8)	5.29 ± 2.20	3.00–10.00	4.00
3	20 (6)	9 (2.5)	11 (3.1)	8.20 ± 3.11	4.00–13.00	7.50
4	28 (8)	14 (3.9)	14 (3.9)	10.86 ± 3.83	5.00–18.00	10.50
5	26 (7)	18 (5.0)	8 (2.2)	13.54 ± 4.39	7.00–26.00	12.00
6	19 (5)	4 (1.1)	15 (4.2)	25.11 ± 8.15	10.00–40.00	23.00
7	19 (5)	1 (0.3)	18 (5.0)	29.42 ± 8.29	14.00–46.00	26.00
8	17 (5)	14 (3.9)	3 (0.8)	23.71 ± 5.85	20.00–39.00	21.00
9	13 (4)	9 (2.5)	4 (1.1)	30.77 ± 13.65	7.00–54.00	29.00
10	17 (5)	2 (0.6)	15 (4.2)	46.35 ± 7.25	35.00–58.00	46.00
11	19 (5)	6 (1.7)	13 (3.6)	43.79 ± 6.32	30.00–52.00	46.00
12	15 (4)	11 (3.1)	4 (1.1)	43.07 ± 9.05	17.00–58.00	46.00
13	20 (6)	6 (1.7)	14 (3.9)	50.65 ± 5.45	38.00–58.00	52.50
14	21 (6)	10 (2.8)	11 (3.1)	51.24 ± 5.90	37.00–58.00	54.00
15	22 (6)	12 (3.4)	10 (2.8)	53.23 ± 6.04	37.00–58.00	56.50
16	21 (6)	4 (1.1)	17 (4.8)	56.05 ± 3.58	45.00–58.00	57.00
17	15 (4)	8 (2.2)	7 (2.0)	56.87 ± 1.78	52.00–58.00	57.00
18	8 (2)	1 (0.3)	7 (2.0)	57.00 ± 2.65	50.00–58.00	58.00
Total:		169 (47.3)	188 (52.7)			

AIMS test results were classified based on result interpretation guidelines (7). Using percentile grids and interpretation guidelines, results were divided into 2 groups:

- Normal – 5th percentile and above
- Low – below 5th percentile

### 3.2 Assessment of validity and diagnostic accuracy

Validation of the Test of IDLA accuracy, compared to the recognized gold standard that is AIMS, allows for precise understanding of its effectiveness and potential clinical applications. Consequently, a series of statistical methods were applied, and the results of the conducted analyses are discussed below.

To determine the direction and strength of the correlation between tests, Spearman’s rank correlation coefficient (rho) was applied. Results are presented in the Table 4.

**Table 4:** Spearman’s rank correlation coefficient (rho).

Parameter	Value
Spearman’s rank correlation coefficient (rho)	0.72
Significance level	*p* < 0.0001
95% Confidence interval for rho	0.664–0.765
Test sensitivity	0.173

Statistical analysis comparing the results of the Test of IDLA with the reference AIMS test demonstrated a significant, positive correlation with Spearman’s rank correlation coefficient equal to 0.72 (rho = 0.72). This value indicates a strong correlation between the results of both tests, suggesting that the Test of IDLA can effectively reflect reality estimated using the gold standard of AIMS. The significance level *p* < 0.0001 confirms that the observed relationship is statistically significant and is not a result of random deviation. Additionally, the 95% confidence interval for rho, ranging from 0.664 to 0.765, demonstrates high precision of the correlation estimation and with 95% certainty supports a conclusion that the true value of the correlation coefficient in the population falls within this range.

**Figure 4:**
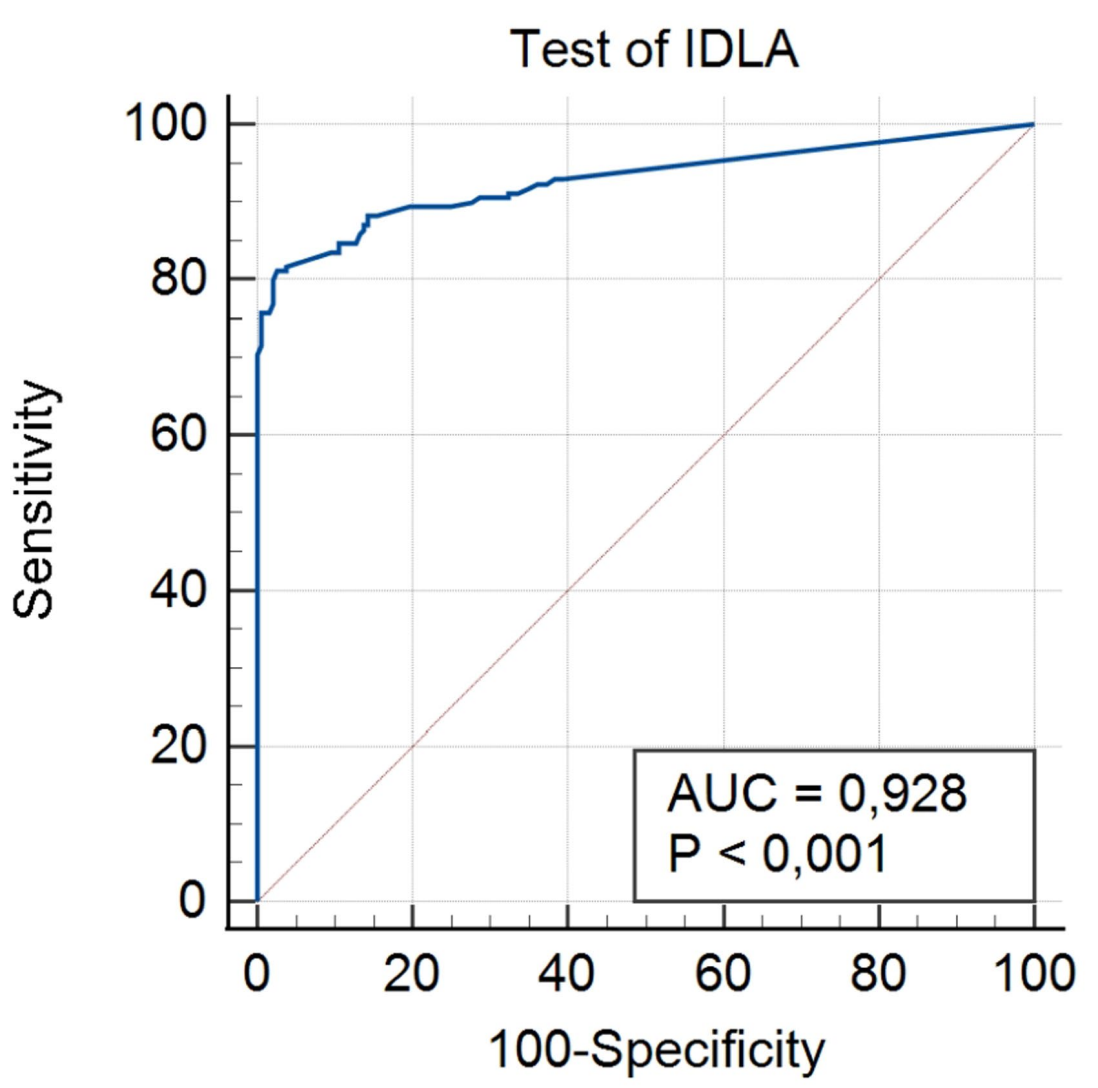
ROC curve.

To conduct further analysis, the cutoff point was estimated. For this purpose, the ROC curve analysis was applied (Figure 4). The dependent variable was the result (Low/Normal) of the AIMS scale. The predictor was the Test of IDLA result AI_(1 & 2)_ (domains AI 1 & AI 2). Results are presented in the Table 5.

**Table 5:** Receiver operating characteristic (ROC) curve analysis.

Parameter	Value
Area under the ROC curve (AUC)	0.928
Standard error	0.0147
95% Confidence interval	0.897–0.953
Significance level P (Area = 0.5)	<0.0001

The obtained area under the ROC curve (AUC) value of 0.928 is particularly significant for the Test of IDLA. In medical diagnostics, AUC values above 0.90 are interpreted as indicating very good diagnostic ability of the test (9). This means that the Test of IDLA demonstrates excellent ability to differentiate between children with delayed motor development and children without developmental delays. This value indicates that there is a 92.8% probability that the Test of IDLA will correctly assign a lower score to a person with greater motor deficits, for two randomly selected individuals.

The narrow confidence interval (0.897–0.953) demonstrates high precision of the obtained AUC estimation for the Test of IDLA (9). The low standard error (0.0147) additionally confirms the reliability of the conducted analysis. These parameters indicate that the actual AUC value in the general population falls within this narrow range with 95% probability, which emphasizes the stability and repeatability of Test of IDLA results under clinical conditions.

The significance level *p* < 0.0001 for the null hypothesis (AUC = 0.5) has crucial importance for the Test of IDLA. The low *p*-value unequivocally proves that the diagnostic ability of the Test of IDLA is significantly better than random classification. This means that the results of the Test of IDLA are not random but reflect the actual ability of the test to identify the examined clinical condition.

The presented results indicate that the Test of IDLA can be recognized as a diagnostic tool of high clinical value. With an AUC value exceeding 0.90, the Test of IDLA qualifies for the category of tests with very good clinical utility, which suggests that it can be successfully applied both for screening and diagnostic purposes (9).

Establishing the appropriate cutoff point is a crucial element in the development of diagnostic tests. The choice of cutoff point involves the necessity of finding a balance between sensitivity and specificity of the test. Cutoff point estimation was conducted considering the following costs associated with potential diagnostic errors:

- Cost of false positive result (FP) = 1
- Cost of false negative result (FN) = 2
- Cost of true positive (TP) and true negative (TN) result = 0

For tests with continuous scale (Test of IDLA), the cutoff point represents a compromise between sensitivity and specificity (10). In the case of the Test of IDLA, a higher cost was adopted for false negative results (FN = 2) than for false positive results (FP = 1), which is justified by more serious clinical consequences of overlooking an actual problem (11). The obtained results are presented in the Table 6.

**Table 6:** Estimation of cut-off point.

Parameter	Value
Pareto optimal criterion	≤3.22
Sensitivity	75.74
Specificity	99.47

Current activity indicator (AI) values of the Test of IDLA scale oscillate within the ranges: 0 ≤ AI<1 Very low; 1 ≤ AI<2 Low; 2 ≤ AI<3 Average; 3 ≤ AI≤4 High. The optimal cutoff point was established at the level of ≤ 3.22 and is classified within the “High” range. This situation creates an interpretational problem, because part of the values from the “High” range (3–3.22) indicate a positive result (which implies a potential problem or the presence of a disease). The remaining part of the range (3.22–4) indicates a negative result (normal) (12). Such ambiguity may lead to erroneous interpretation of test results.

Consequently, consideration should be given to introducing a separate, more detailed interpretation (AI_(1 & 2)_) or introducing modifications to the ranges in the Test of IDLA scale: 0 ≤ AI_(1&2)_ < 1 Very low (positive); 1 ≤ AI_(1&2)_ < 2 Low (positive); 2 ≤ AI_(1&2)_ < 3 Average (positive); 3 ≤ AI_(1&2)_ ≤ 3.22 Moderately high (positive); 3.22 < AI_(1&2)_ ≤ 4 High (negative).

**Figure 5:**
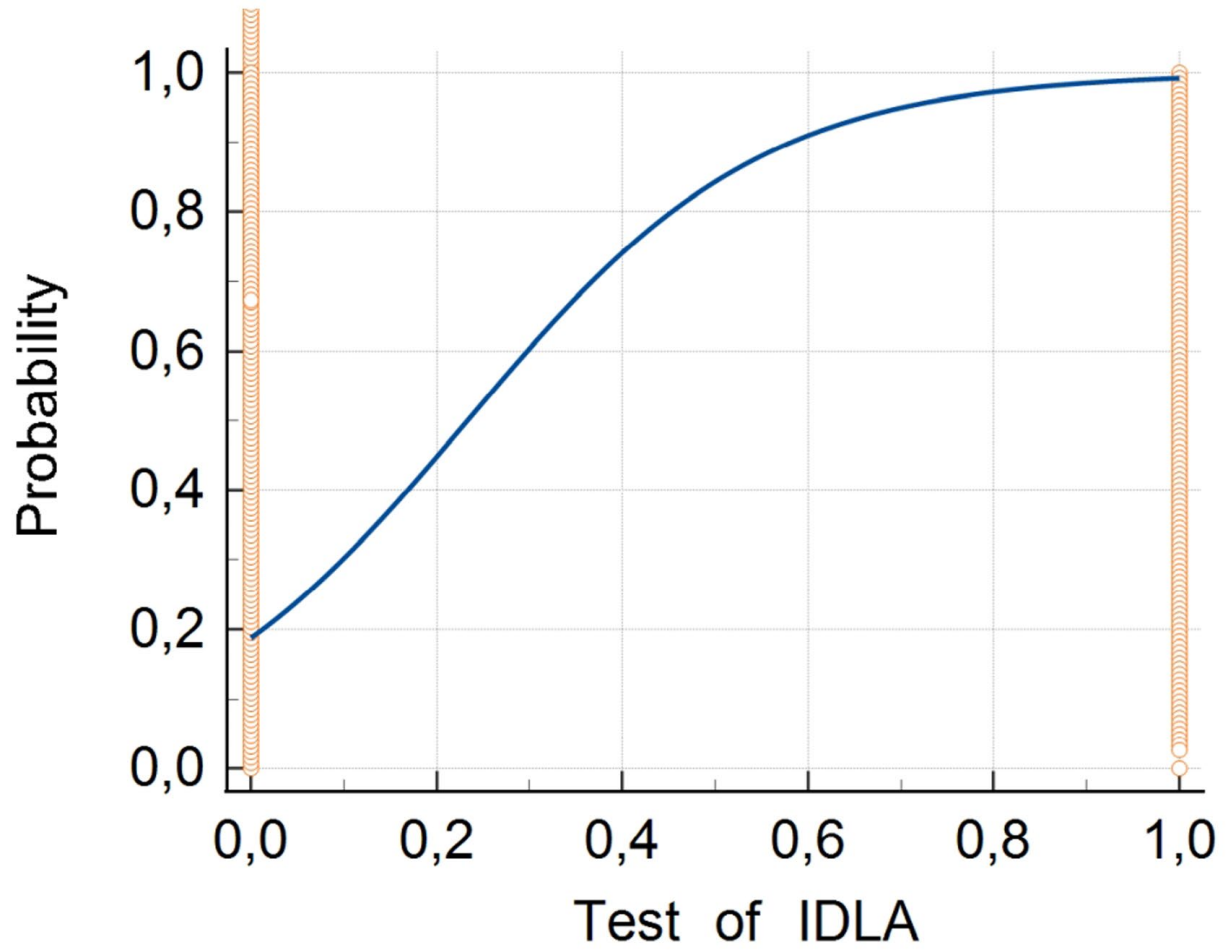
Logistic regression.

This point was selected as optimal in the Pareto sense, meaning that no other cutoff point exists that would improve sensitivity without worsening specificity and vice versa, considering the assumed costs of diagnostic errors. The cutoff point provides sensitivity of 75.74% and specificity of 99.47%, which indicates high effectiveness in detecting delays in motor development. Logistic regression analysis was also performed. Similarly to the ROC curve analysis, the dependent variable is the result (Low/Normal) of the AIMS scale. The predictor is the Test of IDLA result AI_(1 & 2)_ (AI domains 1 & 2). Only one predictor is analysed, no data selection problem occurs, and each introduction method will show the same results. Hence, the “enter” method was applied. Results are presented in the graph (Figure 5) and in the Table 7.

**Table 7:** Logistic regression.

Parameter	Value
Cox & Snell R^2^	0.5271
Nagelkerke R^2^	0.7035
Odds Ratio	583.8049
95% Confidence interval	79.2896–4298.5242
χ2	267.349
*p*	< 0.0001

Results indicate very good model fit to the data, which confirms model significance (χ^2^ = 267.349; *p* < 0.0001). Values of Cox & Snell R^2^ = 0.527 and Nagelkerke R^2^ = 0.704 indicate strong explanatory power of the model. Nagelkerke R^2^ is the preferred measure in logistic regression, as it can achieve a maximum value of 1, unlike Cox & Snell R^2^ (13). The value of Nagelkerke R^2^ = 0.704 suggests that the model explains approximately 70% of variance in AIMS scale results.

The odds ratio (OR = 583.80; 95% CI: 79.29–4298.52) indicates a strong relationship between Test of IDLA results and the AIMS scale. The wide confidence interval suggests considerable uncertainty of this estimation; however, the fact that the lower bound of the confidence interval (79.29) is significantly above 1 confirms the statistical and practical significance of this relationship.

The logistic regression model demonstrates very good fit to the data, and the Test of IDLA demonstrates high concordance with the reference AIMS scale in identifying motor development delays.

Using the χ^2^ test, concordance analysis of two classifications was conducted: AIMS and Test of IDLA. Results are presented in the Table 8.

**Table 8:** χ^2^ analysis.

Parameter	Value
χ^2^	217.518
DF	1
*p*	<0.0001
φ	0.78
Cramér’s V	0.78

The very high χ^2^ (1, *n* = 357) = 217.518 test result indicates a very significant relationship between both classifications, and the *p*-value < 0.0001 indicates significant statistical dependence. The *φ* coefficient and Cramér’s V demonstrate very strong dependence between AIMS and Test of IDLA classifications (14).

To compare face validity and content validity of both tests, anonymous surveys were applied directed to:

- Parents/legal guardians of the child in the case of face validity.
- Physiotherapists working with children aged from birth to 2 years in the case of content validity.

In the survey, participants were asked for:

- Subjective assessment (Positive/Negative) whether the Test of IDLA correctly evaluates child development.
- Subjective assessment (Positive/Negative) whether the AIMS test correctly evaluates child development.

The received responses are presented in the Table 9.

**Table 9:** Assessment of face and content validity.

Parameter	AIMS test score		Test of IDLA score	
	Positive	Negative	Positive	Negative
Face validity *n* (%); SE%	344 (96);1	14 (4);5	343 (96);1	15 (4);5
Content validity *n* (%); SE%	49 (98);2	1 (2);14	49 (98); 2	1 (2);14

Results from the analysis of the received responses show that both tests are perceived as very good diagnostic tools and are characterized by the same face validity and content validity.

## 4 Discussion

Construct and concurrent validity of IDLA in the domains of postural control and motor control in infants were assessed by comparing its results with AIMS scale assessment results, for accurate identification of developmental delays. The decision was made to conduct comparative validation of the postural assessment sheet and motor assessment sheet of the IDLA test, due to the fact that deficits in postural control and motor control are among the most frequently observed abnormalities in clinical physiotherapeutic practice and may significantly delay the process of reaching each consecutive stage in a child’s psychomotor development (15, 16).

The obtained results confirm high concordance between both scales (IDLA and AIMS) in classifying infants in terms of psychomotor development. This concordance is primarily determined by the high value of Spearman’s rank correlation coefficient = 0.72. Additionally, Cramér’s V = 0.78 and χ^2^ (1, n = 357) = 217.518 indicate a very strong relationship between both scales. Based on this, it was determined that the Test of IDLA demonstrates validity and specificity at a sufficiently high level to recognize its psychometric properties in terms of construct validity as comparable to the psychometric values of the AIMS scale.

Spearman’s rank correlation values between 0.70–0.80 were obtained in validation studies of diagnostic tests AIMS, PDMS-2, TIMP, which indicates a strong monotonic relationship between test results, is significant for their reliability and diagnostic validity and demonstrates high concordance and utility in clinical practice (17–19).

Partial validation of the Test of IDLA, encompassing postural control (AI 1) and motor control (AI 2) modules, demonstrated extremely promising diagnostic properties of the instrument. The area under the ROC curve (AUC) of 0.928 (95% CI: 0.897–0.953) qualifies this instrument for the category of tests with “excellent diagnostic accuracy” (9). This value exceeds typical results obtained by recognized scales, such as AIMS (AUC 0.89–0.92) (19–22). This indicates that the Test of IDLA has substantial/immense potential in screening identification of neurodevelopmental disorders.

A fundamental stage in the construct and concurrent validity of the Test of IDLA was determining the optimal cutoff point. According to other studies, cut-off points are established and their effectiveness compared based on the ROC curve analysis, among other methods. The cutoff point represents an established threshold value of the result that allows for the classification of examined children into specific categories of normal development, or risk of developmental delays. This is performed in order to establish a practical interpretation of test results and assess its diagnostic properties (19, 23). According to reports by other authors, higher sensitivity and, consequently, lower cutoff points are preferred in screening studies so as not to overlook children at risk of neurodevelopmental delays, at the cost of a greater number of false positive results. In clinical diagnostics, the cutoff point may be more restrictive in order to limit the number of erroneous diagnoses (24–26).

In the study by Burger et al., the diagnostic value of the AIMS scale was compared with BSID-III (Bayley Scales of Infant Development-III) in children aged 6 months, and the predictive value of AIMS for motor delays at 18 months was assessed. After the ROC analysis, a new cutoff point was proposed that improved sensitivity to 63.6%, with acceptable specificity (81.6%) (27). Narrative and systematic reviews confirm that cutoff points are widely used in scientific research; however, their effectiveness may vary depending on population and child age (23, 28). In the work by Göthner et al., it was demonstrated that AIMS percentile values may not be fully transferable between different countries, which affects the effectiveness of cutoff points and emphasizes the need for local validation (28). Validation studies of the HINE scale suggest that different cutoff points have a high predictive value for diagnosing motor disorders and cerebral palsy, and their effectiveness has been confirmed in comparative analyses with MRI and other instruments (29, 30).

Cutoff points in infant motor scales (including BSID-II, BSID-III, AIMS, HINE, PDMS-2) have a proven predictive value, which has been confirmed in studies with control groups as well as using advanced statistical methods (31–33). Cross-sectional studies in various countries (including Norway and Greece) confirm the applicability of diagnostic scale cutoff points in assessing motor development in infants and indicate the necessity of considering population differences when interpreting results (28, 34).

In light of the above, determining the psychometric parameters of the Test of IDLA for the Polish population appears justified. The optimal cutoff point was established at the threshold of ≤3.22. On a 0–4 scale, it achieves a compromise between sensitivity (75.74%) and specificity (99.47%). Such configuration minimizes the risk of overdiagnosis in high-risk populations (35, 36). It is worth emphasizing that the adopted value of 3.22 is located in the upper part of the activity level according to the original IDLA classification, which requires revision of existing interpretive ranges.

An innovative approach to therapy monitoring, the IDLA – PM subscale, was proposed:

$$\mathrm{AI}\;\mathrm{PM}=\frac{\left(\mathrm{AI}\;1+\mathrm{AI}\;2\right)}{2}$$

And the following result interpretation (a positive result indicates that a psychomotor developmental delay was detected): 0.00 ≤ AI PM < 1.00: Very low (positive); 1.00 ≤ AI PM < 2.00: Low (positive); 2.00 ≤ AI PM < 3.00: Average (positive); 3.00 ≤ AI PM ≤ 3.22: Moderately high (positive); 3.22 < AI PM ≤ 4.00: High (negative).

The above proposal fills a significant gap in monitoring therapy progress in children classified below developmental norms. Unlike AIMS, which focuses on detecting deviations from patterns, IDLA–PM allows for monitoring of subtle changes in daily functioning, which is crucial for personalizing interventions in patients with psychomotor deficits. The AIPM indicator also enables measurement of the rate of functional improvement in infants with psychomotor developmental deficits undergoing therapeutic interventions. The instrument compensates for the lack of AIMS sensitivity in results classified as below norm, enabling precise tracking of effects of implemented physiotherapeutic interventions.

Based on achieved results and drawn conclusions, a comparative analysis between AIMS and IDLA (AI 1 & AI 2) scales was developed, and the IDLA–PM subscale was proposed (Table 10).

**Table 10:** Test comparison.

Parameter	IDLA–PM	AIMS
Diagnostic purpose	Assessment of therapy progress and evaluation of normal development	Assessment of normal development
Target population	General population	General population
Time needed	15–20 min	30–45 min
AUC value	0.93	0.89–0.92
Practical application	Therapy monitoring diagnostics	Diagnostics
Cost	Free	Paid assessment forms

The IDLA–PM subscale focuses on two key domains (postural and motor control), allowing the creation of a specialized screening and therapeutic instrument. AIMS, conversely, does not allow tracking specific therapy progress in children with deficits, making it impossible to use as an instrument which conveys the information about therapeutic intervention progress.

IDLA–PM, as an instrument which meets the criteria for high diagnostic accuracy and low implementation cost, aligns with Lancet Commission on Diagnostics (37) recommendations regarding improving accessibility to early diagnosis of developmental disorders. Short test execution time (15–20 min) and applicability in primary healthcare conditions make IDLA–PM a particularly valuable instrument in healthcare systems, contributing to shortened functional diagnosis time, therapeutic pathway optimization, and improved long-term functional outcomes in at-risk children.

The study’s strength lies in its methodology and research sample. The study encompasses a relatively large sample of 357 infants aged 1 to 18 months, ensuring good statistical power (38). The demographic structure of the sample is well characterized and balanced in terms of gender (175 girls and 182 boys). The study followed a strict protocol described in Section 2.2, consistent with the approved ethical guidelines. The full study protocol is available at: https://doi.org/10.5281/zenodo.18605822. The study utilizes diverse statistical methods to assess psychometric properties of the Test of IDLA. A particularly strong element is the analysis of IDLA diagnostic properties: AUC value at 0.928 (95% CI: 0.897–0.953). This qualifies the instrument for the category of tests with “excellent diagnostic accuracy,” exceeding results of the recognized AIMS scale (AUC 0.89–0.92) (21). Due to the relatively large and gender-balanced sample, application of multiple different assessment methods, and the ROC curve analysis results, the study accurately reflects reality, and the drawn conclusions and analyses are supported by data.

A limitation of the study that should be considered and addressed in supplementary research is the partial validation of the Test of IDLA. The lack of data for dexterity and self-care domains prevents complete characterization of the neurodevelopmental profile, which may affect therapeutic decisions in cases of fine motor deficits and self-care difficulties. Therefore, validation of the complete Test of IDLA version, including all four modules, remains an important issue. Determining the reliability of the Test of IDLA also remains a crucial practical challenge. Another limitation of the present study is the absence of reliability data. However, reliability testing, along with the validation of the dexterity and self-care domains, is currently underway as part of the broader research project and will be reported in subsequent publications.

In summary, IDLA and IDLA-PM constitute an instrument of excellent diagnostic value and high clinical utility, particularly in monitoring therapy progress in infants with psychomotor developmental disorders. Preliminary construct and concurrent validity confirm the justification for its implementation in clinical practice, while simultaneously necessitating further validation studies on the complete test version.

## Data Availability

The datasets presented in this study can be found in online repositories. The names of the repository/repositories and accession number(s) can be found: https://figshare.com/s/b49d2646ae99d447916b.
